# Prevalence and Risk Factors of Internet Addiction among Hungarian High School Students

**DOI:** 10.3390/ijerph18136989

**Published:** 2021-06-30

**Authors:** Krisztian Kapus, Rita Nyulas, Zsolt Nemeskeri, Ivan Zadori, Gyorgy Muity, Julianna Kiss, Andrea Feher, Eva Fejes, Antal Tibold, Gergely Feher

**Affiliations:** 1 Centre for Occupational Medicine, Medical School, University of Pécs, 7624 Pécs, Hungary; kapusk@gmail.com (K.K.); ritanyulas@hotmail.com (R.N.); julianna.kiss@shl.hu (J.K.); efchengirl@gmail.com (E.F.); tibold.antal@pte.hu (A.T.); 2 Faculty of Cultural Sciences, Education and Regional Development, University of Pécs, 7633 Pécs, Hungary; nemeskeri.zsolt@pte.hu (Z.N.); zadori.ivan@pte.hu (I.Z.); 3 Department of Chancellory, University of Pécs, 7633 Pécs, Hungary; muity.gyorgy@pte.hu; 4 Szent Rafael Hospital, 8900 Zalaegerszeg, Hungary; fehergergelydr@gmail.com; 5 Hospital of Komló, 7300 Komló, Hungary; 6 Neurology Outpatient Clinic, EÜ-MED KFT, 7300 Komló, Hungary

**Keywords:** internet addiction, adolescent, epidemiology, risk factor, medical condition

## Abstract

Introduction: The extensive availability of the internet has led to the recognition of problematic internet use (so-called internet addiction—IA) mostly concerning adolescents. Aim: Here, we present a study focusing on the prevalence and risk factors of internet addiction in Hungarian high school students, using a questionnaire-based survey. Results: Overall, 3000 paper-based questionnaires were successfully delivered and 2540 responses were received (response rate of 84.6%). A total of 1309 males (mean age 17.6 ± 1.43 years) (51.5%) and 1231 females (mean age 17.5 ± 1.4 years) (48.5%) took part in our study. Internet addiction was detected in 486 (19.1%) students (232 males, mean age 17.6 ± 1.35 years, and 254 females, mean age 17.34 ± 1.37 years) based on the Problematic Internet Use Questionnaire. In a multivariate analysis, age (age of 17, OR = 3.688, *p* < 0.001), family status (living without parents) (OR = 2.091, *p* = 0.034), the size of the household (more than five people per household) (OR = 2.546, *p* = 0.026), spending more than 6 h online (OR = 5.457, *p* < 0.001), and daily time interval (OR = 84.316, *p* < 0.001) were significantly associated with internet addiction. Alcohol use (OR = 10.341, *p* = 0.001), drug intake (OR = 6.689, *p* = 0.001) and musculoskeletal disorders (OR = 3.966, *p* = 0.047) were also strongly associated with IA. Conclusion: A significant proportion of our students suffered from IA, which is associated with substance intake (possible abuse) and musculoskeletal pain in the multivariate analysis. Our study also draws attention to the preventable risk factors of IA, such as working hours or nighttime internet use, number of hours spent online and family surroundings.

## 1. Introduction

The extensive availability of the internet has led to the recognition of problematic internet use (so-called internet addiction—IA). Problematic internet use is usually defined as the problematic, compulsive use of the internet, resulting in significant impairment in an individual’s function in various life domains over a prolonged period of time. It is an umbrella term rather than a single diagnosis (as it includes pathological gaming, online porn addiction, social media addiction, etc.), but the result is the same: problematic internet users are unable to control their online activities to such an extent that it has a negative effect on their lives [1]. The disorder is increasingly prevalent; it may concern about 6% of the whole population based on the systematic review and meta-analysis published by Chen and his workgroup in 2014 [2]. A very recent meta-analysis showed increased prevalence, most likely driven by the increased rate of internet gaming addiction, reflecting the possible role of increasing human–machine interaction [3]. Problematic internet use usually involves adolescents and young adults; its prevalence can be as high as 25–30% in these age groups, as they are among the first cohorts of people to grow up with easy access to mobile devices and social media, experiencing both the advantages and disadvantages [4,5]. Adopting less severe cutoff levels or permissive polythetic classifications, the rate of addiction can be much higher, underlying the importance of the classification scheme used and cultural factors [6]. The difficulty of its recognition is that internet-based technology has improved many aspects of our lives, and it is now an essential part of our everyday routine, including work, private and social life; therefore, many individuals are not aware of its problematic nature or misuse [7].

Despite intensive research, there are no specific criteria for internet addiction. It can be classified as a compulsive–impulsive spectrum disorder based on symptomatology, but it is under considerable research and not included in the recently published 5th edition of the *Diagnostic and Statistical Manual DSM-V* [7,8].

Several scales are available in the detection of this phenomena, such as the Chen Internet Addiction Scale, 20-item Internet Addiction Test (IAT), 18-item Problematic and Risky Internet Use Screening Scale (PRIUSS), and the Hungarian-developed Problematic Internet Use Questionnaire (PIU-Q), which are widely used and validated in different languages [9,10,11,12]. These tests are more similar than dissimilar, containing identical questions, allowing to focus on the impulsive and risky aspects of internet use [13]. Some of them have identified diagnostic cutoff values, and a researcher or clinician is likely to be well served by any of them [13]. However, it is worth mentioning that evidence-based diagnostic criteria of problematic internet use are still not developed.

IA seems to have several risk factors. The most important ones are a younger age at the start of internet use and being male [13,14]. Males have a two- to five-fold increased risk of problematic internet use compared to females, most likely mediated by differences in personality traits (lower self-control, higher impulsivity and sensation seeking) [14,15]. There is a strong relationship among early internet exposure, initial weekly internet use and the risk of IA [16].

Family functioning has also a crucial role in the development of IA. Lack of family support (less time with parents, less affection from parents) or poor parent–adolescent relationships, such as child abuse or neglect, and single-parent families are also potentially indicated in the development of IA, whereas parental monitoring can be preventive; fathers especially have an influential role [13,17,18]. Online activities such as gaming or social activities may serve an escape from difficult family lives. Children and adolescents are motived to be online in part by escapism and the draw of virtual friendships [13]. Problematic internet use is more common in rural areas and among those with low socioeconomic status [19]. Parents with low income (and with a lower level of education) living in small cities or in villages tend to have less time to supervise their children, spending more time with work (or chasing for work) and having more family conflicts [20].

Certain individual personality traits appear to be common among adolescents with problematic internet use. Impulsivity, aggression and hostility are more common among those with IA, especially among internet game addicts [13,21]. Neuroticism, the tendency to feel nervous and to worry, is identified as a potential predisposing factor of problematic users vs. heavily engaged players [13,22]. It is seen that socially inactive people or those who are dissatisfied with their offline interactions tend to use the internet more frequently; a recent publication showed that living with (any kind of) disability may also increase the risk of IA [23].

Apart from the time spent online (spending more and more time online is a tentative indicator of tolerance, the core criteria of dependence), certain online activities are deliberately addictive, such as gaming, online porn/sex (especially for males) and social media use (especially for females) [5,13]. Several time intervals, such as nighttime internet use, also carry higher risk of IA [5,13].

Problematic internet use seems to be associated with several mental and medical conditions [24,25,26]. Based on cross sectional studies, IA is comorbid with anxiety, depression, attention deficit hyperactivity disorder (ADHD), anxiety, and autism as well as substance abuse, such as alcohol or drug intake. Due to the nature of these studies, the link cannot be entirely clarified. Internet addiction may arise from a pre-existing psychiatric problem or substance abuse, or there is a co-existence and finally excessive internet use probably leads to mental issues [13].

Problematic internet use can also lead to malnutrition and eating disorders, as both excessive weight (sedentary lifestyle, eating fast food, avoid cooking and ordering meals) and malnourishment (being online instead of eating or false body image due to influencers or other famous people) can occur [24].

Furthermore, there is a possible link between internet addiction and increased sympathetic hyperactivity, which can be the precedessor of hypertension and cardiovascular diseases. A recent study showed its association with diabetes and musculoskeletal pain in adults, probably as a result of a sedentary lifestyle, postural habits and fast food consumption [7,27].

However, it has to be noted that there were considerable differences in the methodology and outcome measures, and relatively few studies focused on the complexity (taking the vast majority of the abovementioned risk factors and conditions into account) of IA in Hungary and also worldwide.

The aim of our study was to carry out a cross-sectional questionnaire-based study, focusing on the prevalence and risk factors of IA among Hungarian adolescents, including the detailed demographics and risk factors, such as gender, age, family type, daily internet use, purpose of internet use, type of residence, place of stay, parental education, number of household people, smoking, alcohol and drug consumption, diabetes, hypertension, cardiovascular disease, musculoskeletal pain, depression or other psychiatric diseases, and living with disability.

## 2. Materials and Methods

### 2.1. Participants

This cross-sectional study was conducted between April 2019 and March 2020. The study was approved by the Ethical Committee of the University of Pecs (8434-PTE 2020). Consent was obtained from the school authority prior to data collection. Informed consent was signed by participants before fulfilling the survey and was also confirmed by parents or other guardians if they were underage (<18 years in Hungary).

Paper-based anonymous questionnaires were posted for high school students learning in 8 large educational sites in South- and Middle-Hungary, and completed surveys were collected. The names of the included schools are mentioned in the acknowledgement section.

The inclusion criteria comprised being enrolled as a student during the study period, being willing to participate and having signed, informed consent.

### 2.2. Demographics

The included demographics were gender, age, family type (from married parents to children’s home), type of residence (from own house to institution), place of stay (from small village to big town), parental education (low to high) and number of household people (from 1 to 5 or more).

The age distribution was the following: 3.9% 15 years, 19.6% 16 years, 26.7% 17 years, 23.0% 18 years, 17.2% (437) 19 years, 6.96% 20 years, 1.8% 21 years and 0.84% 22 years of age (Table 1).

A total of 57.7% lived in a conventional family (parents married), while 11.0% in a family with parents in a civil partnership. A total of 24.5% lived with a single parent and 3.4% with foster parents (adopted children).

The vast majority of our students lived in a house, 18.6% in a flat and 6.9% in a farm. The distribution of residence was large town for 28.3%, small town for 36.1%, large village for 534 (21.0%) and small village for 370 (14.6%). The sizes of the households were 3 people for 24.8%, 4 people for 34.1% and five people or more for 31.3% (Table 1). The vast majority of the parents graduated from high school (mothers in 1770 cases (66.7%), and fathers in 1562 (61.5%)) (not shown).

### 2.3. Risk Factors and Concomitant Diseases

The risk factors included smoking, alcohol and drug consumption habits (relatively regularly or not). History of diabetes, hypertension, cardiovascular disease, musculoskeletal pain, depression or other psychiatric diseases were recorded as concomitant diseases and history of disability was also noted.

A total of 8.8% took medication regularly, 10.3% tried alcohol, 22.7% were smokers and 9.3% tried taking drugs more or less regularly. A total of 7.9% of the study population suffered from high blood pressure. The most common disability was visual impairment (5.3%). Detailed data can be seen in Table 2.

### 2.4. Internet Use

Daily time spent online, daily time interval and goals of internet use were also collected.

A total of 21.7% students spent 3 h online, and 449 more than 6 h a day. The preferred time online was between 6:00 p.m. and 9:00 p.m., mostly for chatting and listening to music. The detailed data can be seen in Table 3.

### 2.5. Data Collection Instrument

As there are no clear diagnostic criteria for internet addiction, it is highly recommended to measure excessive internet use with a continuous questionnaire [12]. We chose the Problematic Internet Use Questionnaire (PIUQ) because its structure tightly adheres to the proposed diagnostic criteria for internet addiction and was created based on the clinometric and psychometric analysis of Young’s internet addiction test, independently validated by several groups and used in our previous published work [7,28,29,30]. The questionnaire contains 18 items, each scored on a 5-point Likert-type scale ranging from 1 (never) to 5 (always). A confirmatory factor analysis verified the three-factor model of the questionnaire; each subscale contains six items. Obsession subscale refers to obsessive thinking about the internet (daydreaming, rumination, and fantasizing) and withdrawal symptoms caused by the lack of internet use (anxiety and depression) (“How often do you feel tense, irritated, or stressed if you cannot use the Internet for as long as you want to?”). The neglect subscale contains items about neglecting everyday activities, social life, and essential needs (“How often do you spend time online when you’d rather sleep?”). The control disorder subscale reflects difficulties in controlling time spent on the internet (“How often do you realize saying when you are online, “just a couple of more minutes and I will stop?”). Since in this study we focused on the global psychological consequences of internet addiction, we used the PIUQ total score in statistical analyses, which was computed by summing the scores on all the items of the scale. A total score exceeding 41 points suggests internet addiction [7,28].

### 2.6. Process and Data Analysis

After completing the survey, the participants were divided into two groups based on the results of the PIUQ: (1) addicted to the internet, or (2) not addicted to the internet. At first, the demographic data, risk factors, concomitant diseases and internet use habits were compared between the two groups. The data were evaluated as means ± SD (standard deviation) by Student’s t-test or chi square test to detect significant differences among the examined parameters. To clarify the role of different parameters as independent risk factors of problematic internet use, logistic regression analysis was carried out including all the examined parameters (see above). For all odds ratios, an exact CI of 95% was constructed in our study. Data analysis was performed using SPSS (version 22.0, IBM, New York, NY, USA).

## 3. Results

### 3.1. Demographic Parameters

Overall, 3000 paper-based questionnaires were successfully delivered and 2540 responses received (response rate of 84.6%).

A total of 1309 males (mean age 17.6 ± 1.43 years) (51.5%) and 1231 females (mean age 17.5 ± 1.4 years) (48.5%) took part in our study. The baseline characteristics can be seen in Table 1, Table 2 and Table 3.

### 3.2. Prevalence of Internet Addiction

Internet addiction was detected in 486 (19.1%) students (232 males, mean age 17.6 ± 1.35 years and 254 females, mean age 17.34 ± 1.37 years) based on the Problematic Internet Use Questionnaire. Most frequently, students aged 17 were affected (26.8%) (Table 4).

### 3.3. Risk Factors of Problematic Internet Use

Living without parents was significantly associated with internet addiction (living with foster parents, 4.1% in IA vs. 3.1%, or in living children’s home, 2% in IA vs. 0.7%, *p* < 0.05). Households of more than five people were also more frequently associated with internet addiction (15.2% vs. 10.6%, *p* = 0.008) (Table 4).

Internet addiction was associated with more frequent alcohol (13.7% % vs 9.4%, *p* = 0.008), and drug intake (12.5% vs. 8.5%, *p* = 0.011), history of musculoskeletal pain (2.2 vs. 1.5%, *p* = 0.021) and depression were more also associated with problematic internet use (2.4% vs. 1.8%, *p* = 0.003) (Table 4).

Having disability also increased the risk of IA in general (5.1 vs. 1.9%, *p* < 0.001), but there was no difference in the type of disability between the study groups (Table 5).

Being online for six hours or more was significantly associated with internet addiction (9.2% vs. 5.6% in 6 h, and 32.1% vs. 14.3% in > 6 h, *p* < 0.001) (Table 6). 

Among the purposes of internet use, gaming (76.1 vs. 24.8%, *p* < 0.05) was significantly associated with IA, while chatting had a protective role (17.4 vs. 84%, *p* < 0.001) (Table 6).

### 3.4. Multivariate Analysis

In a multivariate analysis age (age of 17, OR = 3.688, p < 0.001), family status (living without parents) (OR = 2.091, *p* = 0.034), the size of the household (more than five people per household) (OR = 2.546, *p* = 0.026), spending more than 6 h online (OR=5.457, *p* < 0.001), and daily time interval (OR = 84.316, *p* < 0.001) were significantly associated with internet addiction. Alcohol use (OR = 10.341, *p* = 0.001), drug intake (OR = 6.689, *p* = 0.001) and musculoskeletal disorders (OR = 3.966, *p* = 0.047) were also strongly associated with IA (Table 7).

## 4. Discussion

Our research is among the most comprehensive studies from Hungary showing the prevalence and risk factors of internet addiction in high school students.

Based on our results, about one fifth of our high school students suffered from internet addiction, which is significantly higher than the estimated overall pooled prevalence of 7% in the general population [31]. However, based on very recent data, IA prevalence can be much higher among adolescents, which is in line with our results [7,17,28,30,31,32,33]. A recent study showed a rate of 15.5% of problematic internet users in a representative sample of 16-year-old Hungarians, which is nearly comparable to our findings [34]. The increasing prevalence can be due to the extensive availability of the internet, which offers more and more applications and options for engagement; social networking have become a dominant way of social life, which may accelerate the rate of IA.

Internet addiction was common both in males and in females, which is in contrast to previous results showing male predominance [31]. Our results could not confirm the hypothesis of gender-related differences in this addictive behavior [35].

Living without parents or living in a family with more than five people were associated with IA. Low social support, insecure attachment style, poor parent–adolescent relationships and lack of affection were previously shown as risk factors of internet addiction. Growing up without parents can be associated with the abovementioned factors which can explain the higher rate of IA in children living without parents. Living in a large family can also be associated with more conflicts, less communication, and lack of attention and parental support, which can also result in IA [31]. These parameters were independent risk factors of IA in a multivariate analysis.

IA was previously shown to be common in people living in rural areas or having parents with lower educational levels, but these results could not be confirmed in our study [33]. Due to previously documented significant differences in the rate of IA based on place of stay, we also included the type of residence into our analysis, but also obtained neutral results.

Only 20% of our study population spent less than 2 h online, and furthermore, 20% used the internet for more than 6 h a day. Increased frequency of internet use was previously shown to be associated with IA; several studies showed a 2 h cut-off time interval as the predecessor of addiction [17,36]. We found that the cut-off value of 6 h or more of daily internet use to be an independent risk factor of internet addiction, which is in concordance with very recent results [37]. Moreover, several time intervals may precede IA, such as being online between 12:00 a.m. and 3:00 p.m., and between 9:00 p.m. and 12:00 p.m. Night-time internet use previously showed a strong relationship with this phenomenon, and our result draws attention to working (or school) hours of internet use as an important and preventable risk factor of IA [37].

Apart from the hours spent online, several applications, such as social media use or online gaming, were also associated with problematic internet use [38]. In our study, internet gaming was significantly associated with IA but chatting (as part of social media use) was protective; however, in a multivariate analysis, they lost their role as significant predictors of problematic internet use.

A recent case report showed the potential connection between mild intellectual disability and internet addiction [23]. Digital techniques and internet use may provide self-expression and anonymity for people living with any type of disability [23,39,40]. In our research, having any kind of disability raised the possibility of problematic internet use (only in a uni-, but not multivariate analysis), although we could not identify any specific disability.

IA was also associated with substance abuse, such as alcohol or drugs and history of depression. The association between IA and psychiatric symptoms is well documented, but the causality is not well understood, as only cross-sectional studies exist [41]. An underlying psychopathology (history of addiction) may precipitate internet addiction or IA may lead to the onset of consequent behavioral abnormalities and mood disorders, or they may enhance each other [42]. IA also increased the possibility of substance intake (possible abuse) or psychiatric disorders in a multivariate analysis.

IA was also associated with musculoskeletal pain. Few studies have showed the association between pain and internet addiction [43,44]. Both sedentary lifestyles and postural habits/long-lasting fixed positions can play a role [43,44]. In a multivariate analysis, significant association between IA and musculoskeletal pain were found, underlying the importance of IA in the development of chronic musculoskeletal pain, which is the leading cause of disability [45].

In general, our study is one of the most comprehensive reports from Hungary, showing the prevalence and risk factors of internet use among adolescents. A significant proportion of our students suffered from IA, which was associated with substance intake (possible abuse) and musculoskeletal pain in the multivariate analysis. Our study also draws attention to the preventable risk factors of IA, such as working hours or night-time internet use, number of hours spent online and family circumstances.

Finally, our article has some limitations. Due to the lack of a standardized methodology and the absence of randomized studies, these issues were under considerable research and have generated controversy and debate among expert researchers, healthcare and non-healthcare professionals.

Although it was a prospective study in nature including more than 2500 students, it was not representative of internet addiction neither in the general nor in the adolescent population. As it was a questionnaire-based survey, physical examination was not carried out and we had no detailed information about the medical history of the study population, such as the type and duration of musculoskeletal pain, etc. The abovementioned limitations may influence our findings. Finally, follow-up was not carried out.

## Figures and Tables

**Table 1:** Included demographics of the study population.

*Study population (number)*	2540
*Mean age (years)*	17.56 ± 1.41
*Men (number)*	1309 (51.5%)
*Mean age (years)*	17.6 ± 1.43
*Women (number)*	1231 (48.5%)
*Mean age (years)*	17.5 ± 1.4
*Age distribution*	
15	99 (3.9%)
16	498 (19.6%)
17	678 (26.7%)
18	584 (23.0%)
19	437 (17.2%)
20	177 (6.96%)
21	46 (1.8%)
22	21 (0.84%)
*Family type*	
married parents	1465 (57.7%)
parental civil partnership	279 (11.0%)
single parent	627 (24.5%)
Fosterer	86 (3.4%)
students living with partner	46 (1.8%)
children’s home	25 (1.0%)
other (none of the above-mentioned types)	12 (0.5%)
*Type of residence*	
House	1857 (73.2%)
Flat	472 (18.6%)
Farm	177 (6.9%)
Institution	28 (1.1%)
other (none of the above-mentioned types)	6 (0.2%)
*Place of stay*	
big town	720 (28.3%)
small town	916 (36.1%)
large village	534 (21.0%)
small village	370 (14.6%)
*Number of household person*	
1	23 (0.9%)
2	226 (8.9%)
3	631 (24.8%)
4	865 (34.1%)
5	503 (19.8%)
>5	292 (11.5%)

**Table 2:** Risk factors and concomitant diseases in the study population.

*Risk factors and concomitant diseases*	
taking any medication regularly	222 (8.8%)
smoker	578 (22.7%)
taking alcohol more or less regularly	262 (10.3%)
taking drugs more or less regularly	237 (9.3%)
diabetes	48 (1.9%)
hypertension	199 (7.9%)
cardiovascular disease	94 (3.7%)
musculoskeletal pain	40 (1.6%)
depression or other psychiatric disease	48 (1.9)
*Disability*	
ADHD	11 (0.46%)
speech disorder	13 (0.53%)
mental disability	7 (0.3%)
hearing disability	18 (0.73%)
visual impairment	135 (5.3%)
walking disability	7 (0.3%)
mental disorder	6 (0.23)
disability > 1	7 (0.3%)

**Table 3:** Internet use in the study population.

*Daily internet use (approximately)*	
1 h	137 (5.4%)
2 h	419 (16.5%)
3 h	551 (21.7%)
4 h	500 (19.7%)
5 h	327 (12.9%)
6 h	157 (6.2%)
> 6 h	449 (17.6%)
*Daily time interval of internet use (multiply answer)*	
between 12:00 a.m. and 3:00 a.m.	353 (13.9%)
between 3:00 a.m. and 6:00 a.m.	266 (10.5%)
between 6:00 a.m. and 9:00 a.m.	417 (16.4%)
between 9:00 a.m. and 12:00 a.m.	290 (11.4%)
between 12:00 a.m. and 3:00 p.m.	358 (14.1%)
between 3:00 p.m. and 6:00 p.m.	978 (38.5%)
between 6:00 p.m. and 9:00 p.m.	1135 (44.7%)
between 9:00 p.m. and 12:00 p.m.	478 (18.8%)
*Goal of internet use (multiply answer)*	
learning/working	1125 (44.3%)
internet gaming	879 (34.6%)
Chat	1817 (71.5%)
community portal (Facebook, Twitter, etc.)	1201 (47.3%)
Matchmaking	109 (4.3%)
Movies	1501 (59.1%)
Music	1763 (69.4%)
other (none of the above-mentioned types)	13 (5.0%)

**Table 4:** Comparison of baseline characteristics of the study subgroups.

	Not Addicted to the Internet (n = 2054)	Internet Addiction (n = 486)
Gender		
Men	1077 (52.4%)	232 (47.7%)
Women	977 (47.6%)	254 (52.3%)
Age (years)		
15 years	79 (3.8%)	20 (4.1%)
16 years	414 (20.1%)	84 (17.3%)
17 years	500 (24.3%)	178 (36.6%) **
18 years	485 (23.6%)	99 (20.4%)
19 years	366 (17.8%)	71 (14.6%)
20 years	152 (7.4%)	25 (5.1%)
21 years	38 (1.8%)	8 (1.7%)
22 years	20 (0.9%)	1 (0.2%)
Family type (%)		
married parents	1175 (57.2%)	290 (59.7%)
parenteral civil partnership	231 (11.3%)	48 (9.9%)
single parent	518 (25.2%)	104 (21.4%)
fosterer	66 (3.2%)	20 (4.1%) *
students living with partner	40 (1.9%)	5 (1%)
children’s home	15 (0.7%)	10 (2%) *
other (none of the abovementioned types)	9 (0.4%)	9 (1.8%) *
Type of residence (%)		
house	1505 (73.2%)	352 (72.3%)
flat	378 (18.4%)	94 (19.4%)
farm	148 (7.2%)	29 (6%)
institution	18 (0.9%)	10 (2%)
other (none of the abovementioned types)	5 (0.3%)	1 (0.2%)
Place of stay (%)		
big town	592 (28.8%)	128 (26.3%)
small town	718 (35%)	198 (40.7%)
large village	436 (21.2%)	98 (20.2%)
small village	308 (15%)	62 (12.8%)
Number of household person (%)		
1	15 (0.7%)	6 (1.2%)
2	192 (9.3%)	37 (7.6%)
3	522 (25.4%)	109 (22.4%)
4	698 (34%)	167 (34.4%)
5	410 (20%)	93 (19.1%)
> 5	218 (10.6%)	74 (15.2%) *

** *p* < 0.001; * *p* < 0.005.

**Table 5:** Comparison of concomitant diseases and substance abuse and internet use in the study subgroups.

	Not Addicted to the Internet (n = 2054)	Internet Addiction (n = 486)
**Concomitant diseases**		
taking any medication regularly	180 (8.7%)	42 (8.6%)
smoker	443 (21.6%)	135 (27.7%) *
taking alcohol	195 (9.4%)	67 (13.7%) *
taking drugs	176 (8.5%)	61 (12.5%)
diabetes	40 (1.9%)	9 (1.8%)
hypertension	159 (7.7%)	40 (8.2%)
cardiovascular disease	70 (3.4%)	25 (5.1%)
musculoskeletal pain	31 (1.5%)	11 (2.2%) *
depression or other psychiatric disease	39 (1.8%)	12 (2.4%) *
**Disability**		
Any	143 (6.9%)	59 (12.1%) **
ADHD	7 (0.3%)	4 (0.8%)
speech disorder	7 (0.3%)	6 (1.2%)
mental disability	6 (0.3%)	2 (0.4%)
hearing disability	11 (0.5%)	8 (1.6%)
visual impairment	103 (5%)	30 (6.1%)
walking disability	6 (0.3%)	2 (0.4%)
mental disorder	3 (0.1%)	3 (0.6%)
disability > 1	7 (0.3%)	1 (0.2%)

** *p* < 0.001; * *p* < 0.05.

**Table 6:** Comparison of internet use in the study subgroups.

**Daily internet use (approximately)**		
1 h	127 (6.2%)	9 (1.8%)
2 h	371 (18%)	47 (9.7%)
3 h	472 (23%)	79 (16.3%)
4 h	420 (20.4%)	79 (16.3%)
5 h	257 (12.4%)	71 (14.6%)
6 h	114 (5.6%)	45 (9.2%) **
>6 h	293 (14.3%)	156 (32.1%) **
**Daily time interval of internet use (multiply answer)**		
between 0 and 3:00 a.m.	295 (14.4%)	59 (12.1%)
between 3:00 a.m. and 6:00 a.m.	211 (10.3%)	56 (11.5%)
between 6:00 a.m. and 9:00 a.m.	323 (15.7%)	94 (19.3%)
between 9:00 a.m. and 12:00 a.m.	221 (10.8%)	69 (14.2%)
between 12:00 a.m. and 3:00 p.m.	262 (12.8%)	95 (19.5%) *
between 3:00 p.m. and 6:00 p.m.	778 (37.9%)	201 (47.3%)
between 6:00 p.m. and 9:00 p.m.	924 (45%)	210 (43.2%)
between 9:00 p.m. and 12:00 p.m.	346 (16.8%)	132 (27.1%) *
**Goal of internet use (multiply answer)**		
learning/working	911 (44.3%)	214 (44%)
internet gaming	509 (24.8%)	370 (76.1%) *
chat	1732 (84%)	85 (17.4%) **
community portal (Facebook, Twitter, etc.)	965 (47%)	236 (48.5%)
matchmaking	81 (3.9%)	27 (5.5%)
movies	1218 (59.3%)	284 (58.4%)
music	1422 (69.2%)	341 (70.1%)
other (none of the abovementioned types)	100 (4.9%)	27 (5.5%)

** *p* < 0.001; * *p* < 0.05.

**Table 7:** Risk factors associated with internet addiction in a multivariate analysis.

Parameter	Odds Ratio	Significance
Age	3.688 (CI: 2.99–4.44)	*p* < 0.001
Living without parents	2.091 (CI: 1.56–3.04)	*p* = 0.034
Household > 5 people	2.546 (CI: 2.02–3.3)	*p* = 0.026
Being online ≥ 6 h	5.457 (CI: 4.97–6.66)	*p* < 0.001
Daily time interval	84.316 (CI: 66.4–98.5)	*p* < 0.001
Alcohol use	10.341 (CI: 7.49–14.37)	*p* = 0.001
Drug intake	6.689 (CI: 5.01–9.2)	*p* = 0.001
Musculoskeletal disorders	3.966 (CI: 2.9–5.23)	*p* = 0.047

## Data Availability

The dataset supporting the conclusions of this article is available on request to the corresponding author.
